# Exploring Ways to Reduce Heavy Drinking by Increasing Hope Among Midlife Women in Australia: Protocol for a Mixed Methods Study

**DOI:** 10.2196/72628

**Published:** 2025-07-24

**Authors:** Paul R Ward, Megan Warin, Sarah MacLean, Belinda Lunnay, Catherine Palmer, Samantha Meyer, Tonda Hughes, Antonia Lyons, Emily Nicholls

**Affiliations:** 1 Research Centre for Public Health, Equity and Human Flourishing Torrens University Australia Adelaide Australia; 2 School of Social Sciences University of Adelaide Adelaide Australia; 3 Centre for Alcohol Policy Research La Trobe University Melbourne Australia; 4 School of Public Health Sciences Waterloo University Waterloo, ON Canada; 5 Center for Sexual and Gender Minority Health Research Columbia University New York United States; 6 Centre for Addiction Research University of Auckland Auckland New Zealand; 7 Department of Sociology University of York York United Kingdom

**Keywords:** alcohol, sociology of hope, breast cancer prevention, midlife women

## Abstract

**Background:**

Alcohol consumption remains a major societal problem, contributing to myriad health conditions. Australian midlife women (aged 45-64 years) consume more alcohol than previous generations of midlife women and more than younger women now. Alcohol poses health risks that are unique to midlife women, including increased risk of breast cancer; 10% of breast cancers result from alcohol consumption and there is no “safe” limit. There is a global gap in knowledge about socially and culturally appropriate interventions for reducing alcohol consumption in these heavy-drinking groups of midlife women.

**Objective:**

The research questions (RQs) driving the study are as follows: RQ1—what are the shared social practices that constitute and connect alcohol consumption in each of the 4 case study groups of midlife women? RQ2—what are the perceptions of women in the case study groups about the “invisible hands” shaping alcohol consumption in their social worlds? RQ3—what are the possible “systems-level” interventions or strategies to counter or oppose the “invisible hands” impacting women’s alcohol consumption? and RQ4—what is the validity, fidelity, and effectiveness of codeveloped critical consciousness interventions on alcohol-related social practices and consumption patterns?

**Methods:**

Our study will identify and implement ways to support the following groups of heavy-drinking midlife women to reduce their alcohol consumption (and their breast cancer risk): (1) women living in regional areas of Australia; (2) lesbian, gay, bisexual, transgender, queer, and other sexual orientation and gender identity women; (3) women living in poverty; and (4) women working in the corporate sector. An expert group of midlife women, termed a women’s thought collective (WTC), will guide all stages of the study. Stage 1 (0-18 months) will collect qualitative data using social media analysis, netnography, photo elicitation, and ethnography to understand the forces oppressing women and shaping their alcohol consumption. We will recruit 50 participants in each of the case study groups. Stage 2 (19-24 months) will involve deliberative dialogue methods with the case study groups (15 participants per group) to identify policy-level changes required to support the reduction in participants’ drinking. Stage 3 (25-36 months) will codevelop alcohol reduction interventions using critical consciousness development and then implement them using a quasi-experimental design. Primary outcomes are readiness to reduce alcohol and actual changes in consumption levels.

**Results:**

This protocol was funded by the Australian Research Council (DP250104494) for 3 years, starting in July 2025. Human research ethics committee approval has been granted (0457).

**Conclusions:**

Expected outcomes include community actions and policy levers for alcohol reduction, addressing the intersecting sociocultural, political, and commercial factors shaping alcohol use among midlife women. The findings will help inform strategies to counter oppressive forces or transform them into sources of hope, encouraging new practices that challenge social norms.

**International Registered Report Identifier (IRRID):**

PRR1-10.2196/72628

## Introduction

### Research Problem, Significance, and Timeliness

Alcohol consumption remains a major societal problem in Australia, contributing to myriad health conditions and costing an estimated AUD $66 billion (Aus $1=US $1.53) a year (only AUD $6.5 billion is recouped in alcohol tax revenue) [1]). The National Alcohol Strategy 2019 to 2028 aims for a 10% reduction in population-level alcohol consumption [1]. Australian midlife women (aged 45-64 years) consume more alcohol than previous generations of midlife women; currently, the alcohol consumption in this group of women is more than other age groups of women [2], which was exacerbated during the lockdowns due to the COVID-19 pandemic [3]. While men consume more alcohol than women, midlife women experience specific gendered risks from drinking alcohol, including an increased risk of breast cancer (the most common cancer in women [4]), with 10% of breast cancers caused by alcohol alone in addition to other known risk factors such as genetic, hormonal, and environmental factors [5,6]. Midlife women who drink alcohol face a triple burden: (1) all midlife women (irrespective of alcohol consumption) are at risk of breast cancer due to their age; (2) midlife women who drink alcohol have an increased risk of breast cancer (a dose-response relationship—every drink throughout life accumulates breast cancer risk [7]); and (3) midlife women in specific sociocultural groups (the focus of our project) face structural disadvantages that increase their likelihood of heavy alcohol consumption, further increasing their breast cancer risk.

Recent national evidence suggests that attitudes toward alcohol are shifting [8]. Australians increasingly support action against alcohol harms and are willing to reduce alcohol consumption. This change could be a response to increased drinking during the COVID-19 pandemic, increased availability of no- and low-alcohol products, and increased engagement with the sober curious movement [9]. This evidence of social change adds impetus to the potential for our project to contribute to reduced drinking among midlife women. The project extends our recently completed Australian Research Council (ARC)–funded Discovery Project (DP) study (DP190103434) that highlighted a need for new research into the social practices of different groups of midlife women who engage in heavy drinking. The term “heavy drinking” refers to levels exceeding the national alcohol guidelines [10] for long-term (>10 standard drinks per week) or single-occasion (>5 standard drinks on one occasion per month) risky drinking. Existing public health approaches aimed at reducing alcohol consumption (relying on the exercise of individual willpower) have had limited effectiveness among midlife women. We will use social practice theory (SPT) to support and empower midlife women to reduce their alcohol consumption and the multifarious negative impacts on their lives. The advantage of using SPT is that it works at the levels of meanings, materials, and competencies to reduce the extent to which women are forced to rely on willpower to reduce drinking.

### Four Heavy-Drinking Groups of Midlife Women in This Proposed Study

To avoid portraying a homogeneous version of “midlife women,” this project will work with midlife women from four different heavy-drinking social worlds: (1) women living in regional centers [11] (towns or small cities outside capital cities); (2) lesbian, bisexual, or queer (LBQ) cisgender women [12]; (3) women living in poverty [13] (earning <AUD $489 per week after tax); and (4) women working in the corporate sector [14] (senior management in large for-profit corporations in male-dominated sectors, such as mining, construction, finance, and IT). We will explore options for reducing alcohol harms with an intersectional lens, considering social class, sexuality, and ethnicity within and across the case study groups.

### Innovations in the Context of Recent International Advances in Knowledge

#### Overview

There is a global gap in knowledge about socially and culturally appropriate interventions for reducing alcohol consumption in heavy-drinking groups of midlife women. Rather than continuing to problematize individual “choices” to drink, there is an urgent need for research that informs solutions addressing the intersecting community and sociopolitical and commercial factors that shape alcohol consumption among different groups of midlife women. For brevity, these multifaceted structural, systemic, sociocultural factors are referred to as “invisible hands” [15,16]. This project will empower groups of midlife women to “see,” understand, reflect on, and resist these invisible hands to reduce alcohol consumption. The theory and methodology in both the *Pedagogy of the Oppressed* by Freire [17] and the *Pedagogy of Hope* by Freire [18] identified hidden or unseen “limit situations” (which act like invisible hands), which act as oppressive forces, shaping, blocking, or preventing certain human actions (and hopes) in oppressed groups. Freire [17,18] argues that for currently oppressed people to move from oppression to hope, researchers need to work with them using critical consciousness development; individuals can then become critically conscious of the invisible hands and can then work toward overcoming them to increase their hopes. Within the context of the proposed study, we will work with 4 different heavy-drinking groups to identify the invisible hands (or limit situations) acting as oppressive forces to maintain their heavy drinking and prevent reduction in drinking. Once this critical consciousness has been realized, we will co-design interventions to reduce their drinking by overcoming the invisible hands.

#### Theoretical Innovation: Extending the Pedagogies of Oppression and Hope by Freire

Building on the concept of critical consciousness development by Freire [17,18], we will work with 4 groups of midlife women to identify the invisible hands of oppression linked to heavy drinking and codevelop interventions to reduce their drinking based on a pedagogy of hope. We have a unique opportunity to co-design and codevelop new alcohol consumption reduction interventions that have relevance, consonance, and specificity with groups of women whose social worlds are embedded with heavy-drinking practices that will build our research capacity as world leaders and our reputation globally to innovate alcohol consumption reduction approaches situated within social frames. The co-design of interventions that are situationally specific within 4 different heavy-drinking social worlds is a key innovation and extension of the original work by Freire [17,18].

#### Conceptual Innovation: Applying SPT and “Social Worlds”

Our project is novel in investigating what chief investigator S MacLean has conceptualized as “social worlds” [19,20] that encourage heavy drinking and shape women’s practices related to alcohol consumption reduction. Conventional approaches, focusing on changing individual alcohol behaviors independent of social contexts, have had limited effectiveness for the 4 case study groups [21]. Our conceptual innovation is to focus on alcohol consumption not as a discrete behavior but as one enmeshed within broader social practices; the sociality of alcohol consumption is central to the proposed study. SPT focuses on the materials, meanings, and competencies required within particular social practices [22] and directly extends the previous research conducted by chief investigators PRW and S MacLean. In SPT, materials (eg, types of alcoholic or nonalcoholic products) have active, agentic roles within practices, enabling other practices (eg, self-care and managing stress) and creating meanings attached to alcohol (eg, alcohol as crutch, comfort, or toxic) [23]. SPT elements are all shaped by oppressive and gendered forces in women’s lives. Central to SPT is the notion that by changing one or more elements in a practice, the entire social practice can be transformed. For example, participants, through developing critical consciousness of the invisible hands that shape the social practice of drinking, could potentially construct new meanings around the value or relevance of alcohol, choose different material elements (ie, socializing at places where heavy drinking is not facilitated), or develop new competencies (eg, skills to slow down drinking or confidently refuse a drink).

#### Methodological Innovation

Rather than implementing more interventions with limited efficacy for behavior change (a norm for public health concerns related to alcohol), we will undertake a multidisciplinary study, including innovative social practice methods, such as ethnography, netnography [24], photo elicitation, WTCs [25]), and deliberative democracy and critical consciousness development, drawing on our expertise as leading international researchers in sociology, anthropology, gender studies, psychology, and alcohol policy. Our methodology has never been used to explore and then codevelop options for alcohol consumption reduction within our 4 case study groups.

#### Policy Innovation

Alcohol consumption reduction techniques that target individuals (eg, persuading behavior change through screening for problematic drinking) require that consuming alcohol be recognized by the individual as a “problem.” However, most (87%) of the Australian drinkers consider themselves to be “responsible drinkers,” although 68% are risky or heavy drinkers [26], leading to alcohol consumption reduction interventions being resisted (ie, not seeing themselves as problematic drinkers) [27]. Our SPT-codeveloped interventions will provide policy makers with evidence-based, innovative ways to support women in recognizing the invisible hands shaping alcohol consumption within their social worlds and empower them to engage in new or different social practices associated with reduced alcohol consumption rather than requiring drinking to be seen as “problematic” and hence interventions to be resisted.

### Key Research Questions

This is a world-first attempt to codevelop interventions examining gendered practices of alcohol consumption (not individual behaviors) as the sites of social analysis and change. Women in 4 different social worlds will be empowered to recognize, through raising their critical consciousness, the invisible hands (oppression) shaping their heavy-drinking practices. Together with women, new interventions will be developed and tested, which aim to either negate the impact of the oppressive forces or change them into forces for hope (raising critical consciousness to adopt new practices that resist norms embedded within social worlds). Participants will be encouraged to consider why drinking practices are normalized within their social worlds and whether or how this could be different by reimagining group social connections through nondrinking or reduced drinking. The research questions (RQs) driving the study are as follows:

- What are the shared social practices that constitute and connect alcohol consumption in each of the 4 case study groups of midlife women? (identification of pedagogies of oppression; RQ1)
- What are the perceptions of women in the case study groups about the invisible hands shaping alcohol consumption in their social worlds? (raising critical consciousness about pedagogies of oppression; RQ2)
- What are the possible “systems level” interventions or strategies to counter or oppose the “invisible hands” impacting women’s alcohol consumption? (identification of systems-level possibilities for pedagogies of hope; RQ3)
- What is the validity, fidelity, and effectiveness of codeveloped critical consciousness interventions on alcohol-related social practices and consumption patterns? (impact of intervention aimed at pedagogies of hope; RQ4)

The project extends a recently completed ARC-funded study (chief investigators PRW, MW, and BL and partner investigator S Meyer) that explored midlife women’s reasons for alcohol consumption [28-30]. Our critical learning from that study is that women across different social worlds experience different social, cultural, and economic pressures to drink and have different capacities and resources to contemplate reduced drinking, with variations according to intersecting sources of oppression based on social class, sexuality, and ethnicity. Therefore, midlife women require different interventions to reshape their bespoke social practices, socially and culturally specific logics for consuming alcohol, and levels of critical awareness of heavy-drinking norms. This established the need for the proposed project. A subsequent study led by chief investigator BL investigated factors supporting women in reducing alcohol consumption [9], revealing that women in the corporate sector engage in wellness movements as a form of self-determination and increased agency to express a critical awareness of alcohol-related harms, enabling alcohol consumption reduction. However, this framing may not be readily available to other groups of women, highlighting the need for tailored responses in different heavy-drinking social worlds.

### Conceptual Frameworks

We hypothesize that being able to recognize, critique, and counteract the “invisible hands” that shape drinking practices hinges on increasing women’s capacities for collective critical realizations about how gender, sociopolitical settings, and regulations (eg, alcohol marketing to women) related to alcohol intersect with heavy-drinking social practices. Our proposed study will draw on and critique the theories and methodologies of Freire [17,18] to understand whether and how women recognize the “invisible hands” (*Pedagogy of Oppression* [17]) that shape their alcohol consumption practices and examine whether and how they can develop a critical consciousness about the “invisible hands” (making them visible) and implement changes within their social worlds to disrupt and reshape drinking practices (*Pedagogy of Hope* [18]). Rather than emphasizing the behaviors of individual women (avoiding blaming or stigma), we will analyze the interconnected gendered social practices (materials, meanings, and competencies) [22] located within “social worlds” [19] that frame heavy drinking in specific social groups and place women at risk of alcohol-related harms. We bring our sociological imagination to extend the work of other scholars [31-33] who also conceptualize “hope” as a sociocultural phenomenon. In a study of residential substance use services, chief investigator S MacLean found that young people’s hope was rooted in social relationships and productive discourses and varied according to the external resources available to them, giving them some greater capacity than others to pursue their “hoped-for futures” [34]. This is critically important because it means that women in the 4 case study groups may require different social, cultural, or economic resources to gain a sense of control over the invisible hands and their imagined futures.

### Theoretical Framework

We will use SPT [22,35], which has been used extensively by chief investigators PRW, BL [13,36], S MacLean [37], MW [38], and partner investigator AL [39] and identified as critically important for examining how alcohol consumption practices develop, persist, and can be changed [40-42]. Our recent ARC-funded study applied SPT to interpret midlife women’s engagement with alcohol consumption reduction [9] (DP190103434), and chief investigator PRW worked on another ARC-funded study (DP210101166) that focused on applying SPT to oral health practices [43,44]. SPT provides a critical approach for examining alcohol practices in heavy-drinking social worlds and focuses on the shared social practices that frame and support heavy drinking [45]. SPT stipulates that change can occur through addressing one or more of the elements that constitute drinking practices or practices that relate to heavy-drinking behavior. For instance, the social practices of “after work drinks” can be altered by shifting what it means to socially connect with colleagues and offering different ways of connecting that do not revolve around alcohol [46]. Overall, SPT provides a powerful framework for understanding how alcohol consumption reduction can be built into the social worlds of heavy-drinking groups.

## Methods

### Ethical Considerations

Ethical procedures and approvals for all aspects of our study have been approved by Torrens University Human Research Ethics Committee (0457). As with all qualitative research, ethical considerations include obtaining informed consent, ensuring anonymity, and maintaining confidentiality. Alcohol consumption could be a sensitive topic. Our previous qualitative interview studies with diverse midlife women (aged 45-64 y) have attuned us to issues pertaining to informed consent, including detailing the potential risks and benefits of the study. Our approach in related studies has been to demonstrate empathic neutrality in our style of engagement.

Each participant will have an agency to decide the terms of their participation and the information they contribute. Other ethical practices include checking the suitability of the approach with women before commencing data collection; allowing women to choose their preferred pseudonym, mode of contact, and preferred medium for data collection (phone, in person, or telecommunication software); outlining assurances of confidentiality; and issuing honoraria to recompense women’s time and resources. Given the success of our previous studies in generating rich understanding and engaging women across long time periods or repeated interviews [13,28,47,48], we will use similar methods in this study.

The online methods involved in our study, including sharing screenshots, digital content, social media analysis, and photo elicitation, entail unique considerations for informed consent and confidentiality. We will use our previous experience designing ethical online or social media–based studies [49], adapting traditional principles of human research ethics (respect, integrity, and beneficence) to guide practice in online research environments. Shared content that includes nonparticipants (eg, photos with friends and social media influencer posts) will be kept confidential to the research team, and any shared content that is included in research outputs will be digitally anonymized (eg, through blurring faces, names, and identifiable places or signs).

Ethnographic fieldwork in our study will be conducted in person or offline but also entails particular ethical considerations, as researchers are involved collectively in a social world (to observe the social practices shaping heavy drinking). Informed consent and confidentiality are complicated procedures, involving safeguarding against assumptions made by researchers about participants during the process of understanding women’s social worlds. The WTC will be directly involved in the process of gaining access to and developing ethical procedures and field note templates for this aspect of our study.

### Research Design

**Figure 1:**
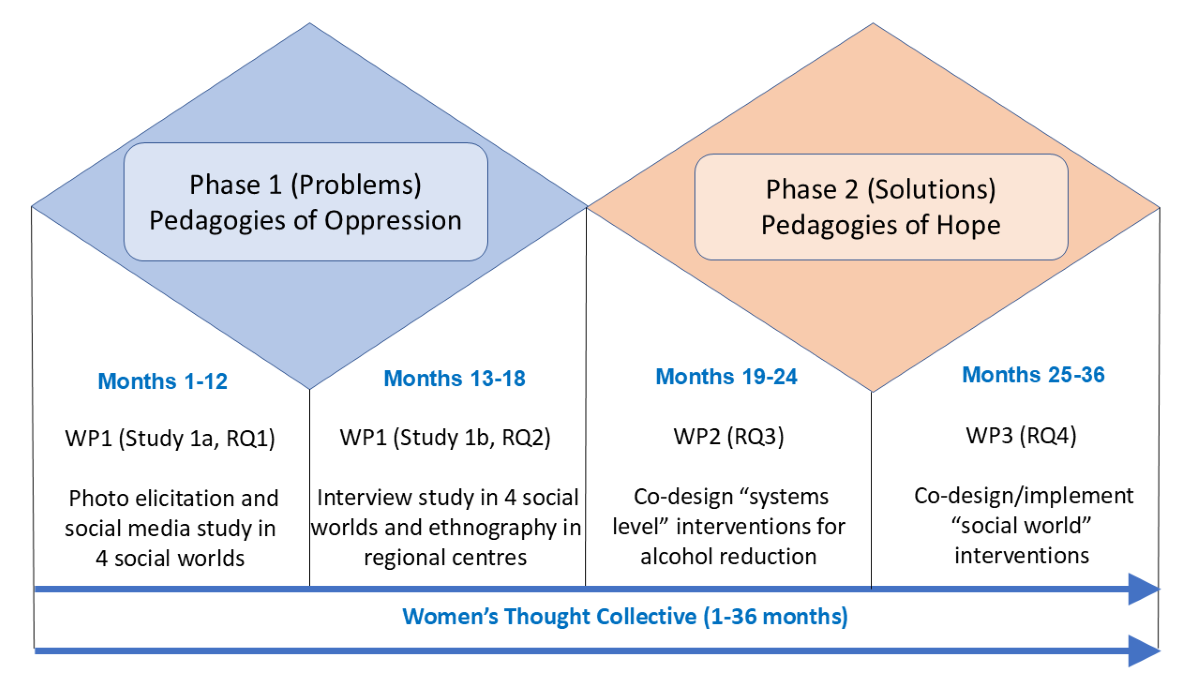
Research design and timelines. RQ: research question; WP: work package.

Our 3-year participatory project follows human-centered design thinking [50] (Figure 1). Within the case study groups, we will recruit samples that enable an intersectional analysis (based on social class, ethnicity, and sexuality) of the invisible hands shaping drinking. We are consciously not focusing on First Nations midlife women because this would require a different research team and a decolonized methodology. However, First Nations women will not be excluded from participating in the case study groups.

The project will be undertaken in 3 work packages (WPs). WPs 1 and 2 will be led by researchers at Torrens (women in the corporate sector and women in regional areas), Adelaide (women in poverty), and La Trobe (LBQ women) universities. WP3 will be led by Torrens University to maintain the fidelity, validity, and reliability of the interventions.

### Project Governance

A project management group (including chief investigators and PIs) will meet monthly to monitor all aspects of the project and identify or manage risks. A stakeholder advisory group (SAG) will be developed to provide ongoing policy advice to the research team, support participant recruitment, and advise on the feasibility of future interventions that may involve their organizations. We will provide early communication of research findings to support their programs (eg, Daybreak app used by Hello Sunday Morning for reducing alcohol consumption). The SAG will meet biannually and include key stakeholders for the case study groups (eg, Chief Executive Women and Hello Sunday Morning for corporate women, Sober in the Country and regional sport assemblies for regional women, Uniting Communities and Women in Poverty Network for women in poverty, and LGBTIQ+ Health Australia and ACON Health for LBQ women) as well as organizations focused on alcohol consumption of individuals and breast cancer support or research (eg, cancer councils and Alcohol and Other Drugs services).

### PhD Student 1: Consumer Engagement and Research Translation Strategies

PhD student 1 (scholarship funded by Torrens University) will collect, manage, and coanalyze data, engaging a WTC that is aligned with participatory action research and feminist hermeneutics or methodologies [25]. The WTC will include 5 representatives of each of the 4 case study groups (n=20) to provide their knowledge, expertise, and experience. The members of the WTC will not be participants in other stages of the study. The WTC will meet quarterly as a group and more often as individuals engaging with the PhD student on specific tasks. The PhD student will work with the WTC to develop, prototype, and test different co-design and research translation strategies. The WTC will provide meaningful and sustained co-design of data collection strategies and coanalysis of data from WP1 and WP2 (eg, coanalysis of anonymized data from some interviews, netnography, and ethnography). The WTC will also generate “ground truth” data from the dialogue groups and codeveloped interventions (WP3) in terms of feasibility and real-world applicability. We developed and piloted the WTC method with midlife women in our recent ARC DPs, garnering numerous additional insights into recruitment, data collection, data analysis, and real-world interpretation [25].

### PhD Student 2: Ethnography in Regional Centers in South Australia

PhD student 2 (scholarship funded by Torrens University) will take responsibility for data collection and analysis for the women living in regional centers for WP1 and WP2. In addition, PhD student 2 will undertake ethnographic data collection in selected regional areas, including wine growing areas in South Australia (eg, Barossa Valley and Clare Valley; local to PhD supervisors and chief investigators PRW, MW, and CP), to understand the invisible hands and situational issues for midlife women in social worlds saturated by alcohol-related industries (eg, viticulture, wineries, distilleries, agricultural suppliers, hospitality, and tourism, etc) and alcohol sponsorship or donations to community sports clubs or associations where alcohol is sold and offered as raffle prizes to raise revenue for club activities and sustainability.

### WP1 (RQ1 and RQ2; 0-18 Months)

#### Overview

Ethnography, netnography, photo elicitation, social media analysis, and qualitative interviews will be used to explore the factors that impact women’s possibilities for alcohol consumption reduction within their social worlds. Midlife women will share how, where, and when heavy-drinking practices persist and identify the “invisible hands” that “push” them to drink and “pull” them away from reducing alcohol consumption and how drinking practices embedded in each of their social worlds facilitate heavy drinking. With the view to inform future interventions, they will be asked to describe protective (hope-based) factors that “shield” against “invisible hands” and their ideas on how, why, and where shields improve capacities for alcohol consumption reduction. We will target localized cultures and practices within workplaces, leisure spaces, and communities with the view of identifying new and actionable competencies in relation to alcohol consumption reduction within each social world.

Recruitment strategies will be tailored for each group (based on advice from the SAG and WTC), although we will be guided by previous successful approaches to recruitment, including targeted Facebook campaigns and snowball sampling techniques [51]. The research team will use their long-term relationships with organizations and networks to support recruitment: for women living in poverty through state and national networks (eg, Women in Poverty Network [chief investigator BL is a board director] and National Council for Women); for LBQ women through LGBTIQ+ Health, Thorne Harbour Health, and ACON Health; for women in regional areas through regional sport assemblies and sport or community associations; and for women in the corporate sector through Chief Executive Women and Hello Sunday Morning. All participants will be aged ≥18 years and will self-report “heavy” or “moderate” drinking.

#### Data Collection for Netnography, Photo Elicitation, and Social Media Analysis (RQ1; Study 1A)

We will recruit 50 participants for each case study group across Australia (n=200) who will create and upload screenshots to a study portal (n=5-10; a minimum of 5) of social media content that they perceive as reinforcing alcohol norms within their social worlds. In addition, participants will be asked to take photographs (and upload to the study portal) of “invisible hands” in their local built environment, community, or workplace that they perceive are social worlds that promote heavy-drinking practices or act as social worlds that promote practices that do not involve alcohol consumption. Participants will be provided with examples of relevant content. chief investigators PRW and BL and partner investigator AL are experienced in netnography and social media analysis [49,52,53], which will enable a meaningful yet manageable volume of content for analysis [54]. Participants will record where and when they saw the image (eg, social media platform or location in their local area, day or night, and day of week) and rate the content in terms of personal appeal, impact, and intention (method undertaken by chief investigator PRW [54]).

#### Data Collection for Qualitative Interviews (RQ2; Study 1B)

This stage will use qualitative methods to explore heavy drinkers’ openness to reducing alcohol consumption among the 4 case study groups. Research assistants and PhD student 2 will conduct in-depth interviews with approximately 30 participants from each case study group (n=120), noting that sampling will continue until saturation is reached [55]. Previous experience suggests this sample size will allow for observable patterns within the data for each case study group. Some moderate drinkers from each group will be recruited alongside heavy drinkers for variability to triangulate observations on the social context of drinking within their particular social world [19]. Interviews will be conducted online and last approximately 1 hour. Interviews will probe for knowledge, attitudes, beliefs, and practices around possibilities for reducing alcohol consumption (particularly where drinking is habitual and tied to social settings, identities, or environments); awareness (critical consciousness) of the invisible hands (social, cultural, economic, and community factors) shaping their drinking; and what alternatives to drinking alcohol they perceive to be available in their social worlds. Data analysis will use NVivo (Lumivero) and follow the well-used 3-step progressive method of precoding and conceptual and theoretical categorization by chief investigator PRW and partner investigator S Meyer [56]. Chief investigators and partner investigators will meet regularly in person during this stage to check for agreement in coding (each cocoding a specific set of transcripts) to improve explanatory rigor. Together, they will collate the data for use in WP2.

### WP2 (RQ3; 19-24 Months)

Deliberative dialogue groups (4 groups; 15 participants per group) will be used to identify “systems level” (eg, invisible hands not immediately amenable to individual action) drivers of alcohol consumption in the 4 case study groups (note that invisible hands amenable to individual action will be the focus in WP3). Dialogue groups will run separately for each of the 4 case study groups, with each dialogue group comprising midlife women from the case study group (recruited from participants in WP1) along with representatives from relevant stakeholders (upon advice from the SAG and WTC). Chief investigator PRW has extensive experience with deliberative democracy methods [57-59], and a group size of 15 will allow both depth of deliberation and meaningful dialogue. Two weeks before the dialogue groups, materials will be distributed to orient participants to the findings from WP1 (analyzed by the researchers and the WTC). Each dialogue group will meet online over a 2-day period (usually on weekends to increase recruitment and attendance, as in the National Health and Medical Research Council study by chief investigator PRW [60]) to consider the evidence from WP1 and discuss feasible options for “system level” changes for alcohol consumption reduction in the respective social worlds. Deliberations will focus on how to shift meanings, materials, and competencies (elements of SPT) to reduce alcohol consumption. Participants will discuss their understanding of the “systems level” invisible hands shaping their alcohol consumption, which are likely to be different for each case study group. With input from the stakeholders, they will then discuss ways to reduce or negate the impact of the invisible hands. Chief investigators, partner investigators, and stakeholders will participate in the deliberative process to combine empirical knowledge with women’s real-world examples of practices on what “works” in harm reduction to ensure the suggested strategies are feasible. Once the invisible hands are identified, the dialogue groups will codevelop policy, practice, or advocacy strategies required to reduce or challenge their oppressive forces and move toward a pedagogy of hope (and reduced alcohol consumption). The outcomes will be codeveloped options for intervention at “systems levels,” which could include alcohol policy interventions (eg, reducing price of no or low alcohol products and regulating feminized alcohol marketing) or broader social policy interventions (eg, domestic violence support, advocacy around inclusion, and antidiscrimination).

### WP 3 (RQ4; 25-36 Months)

Interventions will be codeveloped and implemented (using a quasi-experimental design), aimed at reducing alcohol consumption (moving from oppression to hope) in each of the 4 case study groups. The key findings and analysis from WP1 and WP2 will form the basis for WP3. We will codevelop, implement, and test 1 intervention per case study group, evaluating the impacts on women’s critical consciousness development, their preparedness to question heavy-drinking social norms, their ability to have critical conversations about alcohol, and their readiness to change alcohol consumption patterns and make reductions in drinking. WP3 will be based on the original conception and implementation of critical consciousness presented in the *Pedagogy of the Oppressed* by Freire [17], which has been updated, validated, and tested by chief investigator PRW [61-63]. This will enable women to have critical conversations about alcohol with others in their social worlds (a competency in SPT), which is part of a renewed and less oppressive alcohol-related social practice. The WTC will provide expertise on adjustments required to facilitate new social practices within the case study groups. Participants will be recruited from WP1 and WP2. In line with the previous application of the critical consciousness intervention by chief investigator PRW [61-63], we will recruit 15 participants for each of the case study groups. This sample size enables sufficient variance within each group alongside a manageable number for the co-design and implementation of the intervention. While this sample size will not enable statistical analysis of the efficacy of the interventions, the purpose of this stage is to develop interventions and assess their feasibility, fidelity, and acceptability. Subsequent studies can then implement and test interventions, with sample sizes that are adequately powered for efficacy.

### Co-Design of the Critical Consciousness Interventions

The 4 case study groups will meet separately with the research team to identify and prioritize potential interventions within their social worlds. This will occur over 8 fortnightly critical consciousness development sessions, each with its own aims, processes, materials, and outcomes (chief investigator PRW has published details of these 8 sessions [61-63]). Each session builds on the previous session, requiring participants to identify the invisible hands that shape their alcohol consumption (eg, to raise their critical consciousness), reflect on the amenability to change specific invisible hands, and ultimately identify and prioritize (as a group) the invisible hands that, if changed, are most likely to reduce their alcohol consumption. In taking the SPT approach, the co-designed interventions will focus on responding to elements of practice (ie, materials, competencies, and meanings) and their temporal connections. As an example for the “women in the corporate sector,” interventions will address social practices of drinking in the corporate social world rather than individual drinking behavior. Interventions will target elements of practice that combine the corporate site (corporate spaces and alcohol products—materials), the know-how required to perform in this environment (gendered expectations in corporate culture—competence), and the significance of shared drinking practices in the corporate sector (managing “nonwork” caring responsibilities alongside “work” after-hours drinking—meaning). Identifying and intervening with the invisible hands that reflect tensions between corporate work cultures, gendered caring responsibilities, and bonding through alcohol consumption provides opportunities for changing social practices of drinking.

### Implementation of the Interventions

Within each case study group, one intervention will be identified during the previous stage (critical consciousness development) that could help challenge some of the invisible hands that encourage heavy drinking or discourage reduced drinking. The intervention will then be implemented for 6 months, which is a reasonable time to identify changes (initial and sustained) in alcohol-related practices (reductions in alcohol consumption, substitution with nonalcoholic beverages, and changes in other social practices to sustain reduced drinking). Using participatory action research, participants in each of the case study groups (n=15 per group) will implement the intervention within their social world and report on the process and outcomes of the intervention. A process evaluation will be undertaken by collecting qualitative data on the barriers and enablers to implementing the intervention within their social worlds. Throughout the 6 months of the intervention, each case study group will meet monthly to provide feedback to the group and discuss the fidelity, acceptability, and feasibility of the intervention and potentially refine the intervention based on the dynamics of their social world. In addition, individual interviews will be conducted with all participants (before, during, and after the intervention) to understand any context-specific issues in relation to the suitability of the intervention within their social worlds and their level of critical awareness of the factors that impact their drinking, noting issues they perceive as modifiable and more challenging or complex. To measure the effectiveness of the interventions, we will collect quantitative data on both readiness to change alcohol consumption and changes in alcohol consumption (using the validated Alcohol Use Disorders Identification Test) at the study outset, during the intervention development stage, and 6 months after the intervention.

## Results

This research protocol was funded by the ARC as part of their DP scheme (grant ID DP250104494). The study has been funded for a 3-year period, starting in July 2025.

## Discussion

### Enhanced National and International Collaboration

This collaborative study brings together an exceptional multidisciplinary team from Australia and internationally (Auckland, New Zealand; Columbia, United States; York, United Kingdom; and Waterloo, Canada). The research team is a bespoke blend of high-performing, internationally renowned researchers experienced in translating research into policy. We formed a new multidisciplinary (sociology, anthropology, psychology, gender studies, and alcohol studies) and multi-institutional (national and international) team. We are currently undertaking a multicountry cross-sectional survey with LBQ women in Australia (chief investigators PRW, S MacLean, and BL), New Zealand (partner investigator AL), Scotland, and the United States (partner investigator TH) to understand barriers to and facilitators of alcohol consumption reduction, which will feed into the case study group of LBQ women. We have built a team that will develop capacity for the next cadre of exceptional researchers, and we will identify opportunities to build EMCRs research track records and capacity in research translation throughout and beyond the project, with mentoring from senior researchers. Our international research collaborations will enable sister studies to be undertaken in the United States, Canada, New Zealand, and United Kingdom, growing our global profile.

### Key Benefits of the Study

At a theoretical level, our innovative synthesis of the “invisible hands,” Freire’s pedagogies of oppression (1993) and hope (2021) and SPT will advance calls to “curb our culture of intoxication [1]” by pushing past limitations of individual behavior approaches. Our entirely new coupling of pedagogies of oppression and hope with SPT leverages our multidisciplinary expertise across sociology, anthropology, gender studies, psychology, and health promotion and will advance knowledge around “hope” for social change. Benefits will include new applications of critical consciousness, new applications of pedagogy of the oppressed and pedagogy of hope to a contemporary example with real-world health and social implications, comparative data across different social worlds about the invisible hands acting as oppressive forces against midlife women, and comparative understandings of the shared and different social practices in the social worlds of women in the different case study groups.

The proposed study will provide critically important new ways to support alcohol consumption reduction among 4 case study heavy-drinking groups, aligned with the Australian National Alcohol Strategy 2019-28 [1] and the World Health Organization Global Alcohol Strategy [64]. The interventions are more likely to be impactful because women themselves (the case study groups and the WTC) will codevelop them to align with the values and beliefs in their social worlds. A direct and immediate benefit of our empirically driven project is that it draws attention and responds to the “invisible hands” forming alcohol practices among women as points for immediate intervention (a pedagogy of hope).

The interventions will have benefits at 2 levels: community-level actions that are immediately actionable—the results from WP3—and policy or practice levers (results from WP2) readily available for action when “political will” can be harnessed, preparing us to strategically respond to these openings for advocacy.

### Potential Limitations of the Study

This is a methodologically complex study, applying numerous sociological theories and implementing multiple qualitative and quantitative research methods. Although our research team has excellent professional networks and extensive experience in undertaking research with each of the case groups, a potential limitation is that we may not recruit enough participants in each of the case study groups. This potential limitation will also be reduced because we will convene a WTC, which will support the recruitment and retention of participants in the study. We also have a plan for recruitment from one stage of the research to the next, with a proportion of participants in stage 1 being recruited into stage 2 and then a proportion of those being recruited into stage 3. This increases the feasibility of successful completion and outcomes from the study. A further potential limitation relates to the implementation of the interventions in stage 3. The actual details of the interventions cannot be known until we have completed stage 2, but the uncertainty of the types of interventions is a potential limitation.

### Anticipated Outcomes of the Study

The study will contribute to mobilizing women to develop a more critical attitude toward alcohol consumption within the relationships, contexts, and spaces (eg, their “social worlds”) where heavy drinking is enabled. Our focus on community-level actions and policy or practice levers for change will contribute to social, economic, and public benefits, extending beyond personal health outcomes to those positively benefiting society. This project will provide evidence for targeted disinvestment in approaches with limited effectiveness in reducing alcohol-related harms for heavy-drinking groups of midlife women. We aim to provide a template for social change toward enabling social practices within heavy-drinking social worlds where reduced drinking becomes easier to imagine and realize. Given our international research team, there will be huge potential for developing sister studies in other heavy-drinking social worlds in the United Kingdom, the United States, New Zealand, and Canada.

## Appendix

Multimedia Appendix 1 Peer review report by the Australian Research Council (Australian Government).

## Abbreviations

ARC: Australian Research Council
LBQ: lesbian, bisexual, or queer
RQ: research question
SAG: stakeholder advisory group
SPT: social practice theory
WP: work package
WTC: women’s thought collective

## Data Availability

The anonymized datasets generated or analyzed during this study may be available from the corresponding author on reasonable request (for people to access any of our data, they and authors will need to apply for new ethics committee approvals).
