# Supplementary material for: Exploring Ways to Reduce Heavy Drinking by Increasing Hope Among Midlife Women in Australia: Protocol for a Mixed Methods Study
Source: JMIR Res Protoc. 2025 Jul 24;14:e72628. doi: 10.2196/72628 (PMC12332450; doi:10.2196/72628)
Supplement: Multimedia Appendix 1 [file resprot_v14i1e72628_app1.pdf]

Australian Government
Australian Research Council

Search

Dr Belinda Lunnay

Action Centre / Applications / DP250104494 (Submitted to ARC)

Details | Assessment | Funding | Feedback | Comments | History

Application ID DP250104494

Title From Oppression to Hope: Reducing Heavy-Drinking with Midlife Women

Admin Organisation Torrens University Australia

Investigators

1. *Prof Paul Ward (Chief Investigator)*
2. Prof Megan Warin (Chief Investigator)
3. Prof Sarah MacLean (Chief Investigator)
4. Dr Belinda Lunnay (Chief Investigator)
5. Prof Catherine Palmer (Chief Investigator)
6. Dr Samantha Meyer (Partner Investigator)
7. Prof Tonda Hughes (Partner Investigator)
8. Prof Antonia Lyons (Partner Investigator)
9. Dr Emily Nicholls (Partner Investigator)

Organisations

1. Torrens University Australia (Administering Organisation)
2. La Trobe University (Other Eligible Organisation)
3. The University of Adelaide (Other Eligible Organisation)
4. University of Waterloo, Canada (Other Organisation)
5. The University of York, UK (Other Organisation)
6. Columbia University, New York, USA (Other Organisation)
7. The University of Auckland, NZ (Other Organisation)

Application Summary This project aims to reduce alcohol consumption in 4 heavy drinking groups of midlife women by developing/testing co-designed interventions aimed at changing social practices around alcohol. This project expects to generate new knowledge on the personal, social and cultural drivers of heavy drinking using novel interdisciplinary approaches combining social practice theory, critical consciousness and pedagogies of oppression and hope. Expected outcomes include: community-level actions and policy/practice levers for alcohol reduction; and enhanced capacity for the research team to address the societal impacts of alcohol on the global stage. This should provide significant benefits in terms of reducing alcohol consumption for midlife women.

Application Status Submitted to ARC

Outcome Funded

Australian Government
Australian Research Council

Search

Dr Belinda Lunnay

Action Centre / Applications / DP250104494 (Submitted to ARC)

Details | Assessment | Funding | Feedback | Comments | History

Indicative funding by financial year

2024-25	2025-26	2026-27	2027-28	2028-29	2029-30
$140,134.50	$282,241.00	$243,815.00	$101,708.50	$0.00	$0.00

Indicative funding by calendar year

2025	2026	2027	2028	2029
$280,269.00	$284,213.00	$203,417.00	$0.00	$0.00

If any other special conditions apply to this funding, they are as follows:

- Nil

Action Centre / Applications / DP250104494 (Submitted to ARC)

Details | Assessment | Funding | Feedback | Comments | History

Assessment Details

Application Panel Social, Behavioural and Economic Sciences

Application Panel Rank 2

Application Panel Rating A

General Assessments

Assessor	Carriage 1	Rank	Weighted Rating	Normalised Weighted Rating
General 1 Hide	No	1/38	B	A
**Assessment Criteria Scores and/or Text**<br>**Investigator(s)/Capability (B)**<br>**Project Quality and Innovation (A)**<br>**Benefit (B)**<br>**Feasibility (B)**				
General 2 Hide	Yes	3/34	B	A
**Assessment Criteria Scores and/or Text**<br>**Investigator(s)/Capability (A)**<br>**Project Quality and Innovation (B)**<br>**Benefit (B)**<br>**Feasibility (B)**				

Detailed Assessments

Assessor	Rank	Weighted Rating
Detailed 1 View	1/3	B
Detailed 2 View	1/2	A
Detailed 3 View	1/1	A

Detailed Assessments

Assessor Id	Actions
A	Hide

Investigator(s)/Capability

The project team comprises five CIs, four PIs. This is a strong team, with each of the CIs having relevant expertise to undertake this project to high standards. The principal CI has an outstanding track record, with highly relevant and extensive publications and grants in public health and sociological scholarship. They bring extensive experience in project management, research leadership and mentoring. The other CIs are very highly respected scholars in sociology, gender studies, anthropology and alcohol research, with one of them being a more junior researcher. Each CI has good practice and policy experience, which positions them well to access recruits for the planned interviews and use the evidence generated to create impact. Most of the CIs have worked together previously and are well versed in the different research methods proposed for use in the project. This project also builds nicely from a previous DP grant that CIA held. The team is made up of the researchers in Australia who are best placed to undertake this research and positions them very well to produce scientifically robust, innovative and useful research. My only point is that the time commitment from each CI is quite small - CIA 25% of time, CIB 0.2, CIC 0.1, CID 0.2 and CIE 0.2. 2 PhD students are noted (funded by Torrens University)

Project Quality and Innovation

This project addresses a very important societal issue of heavy alcohol drinking among mid life women and connects it very clearly with current Australian policy direction.

The aim of the project is clear: to investigate the social practices with different heavy-drinking groups of midlife women, with the purpose of identifying socially and culturally appropriate interventions. The research questions are highly appropriate for the aims of the project.

The team will draw on social practice theory, which they are expert in, and make use of Freire's oppression and hope perspective in a very creative way. Using a co-design approach with the different categories of heavy drinking women, the team aims to develop alcohol reduction interventions that are highly relevant and specific to the women. This approach is excellent and offers a way to create interventions that, while reflective of the wider structural social worlds in which people live, respond to the needs of these women as articulated by themselves.

The project will make use of a range of really innovative methods (ethnography, netnography, photo elicitation, Women's Thought Collectives, deliberative democracy and critical consciousness development) that seem very fit for purpose and will elicit exactly the sort of evidence in an appropriate manner that will be necessary to create the types of behaviour change being sought. I have not seen this approach in alcohol studies before and am very excited by it. The team clearly know their way around the theories and methods that they have outlined to deliver on the project.

I can follow and understand the methods proposed for the data collection and analysis and that all seems very fit for purpose. However, there is a lot going on, with a lot of data collection. It would be good to have a much clearer explanation of governance and project management - the PhD students seem to be doing a lot of the heavy lifting in data collection and analysis. Is the project basically a vehicle for two PhDs, supervised by the CIs? It seems that the project would really benefit from a fulltime research fellow and project manager. A researcher is noted in the budget section but I don't see them in the project [illegible]

The team clearly know their way around the theories and methods that they have outlined to deliver on the project.
I can follow and understand the methods proposed for the data collection and analysis and that all seems very fit for purpose. However, there is a lot going on, with a lot of data collection. It would be good to have a much clearer explanation of governance and project management - the PhD students seem to be doing a lot of the heavy lifting in data collection and analysis. Is the project basically a vehicle for two PhDs, supervised by the CIs? It seems that the project would really benefit from a fulltime research fellow and project manager. A researcher is noted in the budget section but I don't see them in the project description?

Benefit

The team have very clearly demonstrated the scholarly and practical benefits of this project. It is a very exciting piece of research, bringing together theoretical perspectives in new ways, using very creative methods that have the real possibility of making change happen. The resulting practical benefits could be enormous for midlife women and society more broadly - reducing heavy alcohol drinking is a very difficult thing to do but the co-designed methods being proposed in this project offer real possibility.

Feasibility

The project is very feasible and good value for money. The focus of the project is tight, with a clear aim (to create co-designed interventions to reduce heavy alcohol use among midlife women) and with well developed guiding research questions. While it is a busy project, with multiple stages and sub projects and methods, the team are well experienced and very capable in delivering this. They have worked together and shown that they can deliver in similar areas of research. They have previously written together and undertaken empirical research together.

C Hide

Investigator(s)/Capability

Investigator capability is very strong. This strength represents four factors: (1) the exemplary track record of lead investigator Ward, who not only has outstanding metrics for his very high productivity, but who has a well established record of powerful conceptual and methodological innovation across a number of areas of health research. CI Ward's capacity for productive and insightful innovation is evident in this proposal. (2) The collective strength of the team that Ward has assembled, which combines researchers from his own Centre, all of whom have good track records (in some cases, such as CI Warin, outstanding) (3) The Partner Investigator team, which is also characterised by good to outstanding trach records and who represent both additional expertise and additional reach and impact for the project and (4) the strength of the connections and collaborations that have already been established among the Investigators, particularly represented in high quality coauthored publications from the group at Torrens.

Project Quality and Innovation

The project addresses the substantial harms known to be generated by moderate to heavy alcohol consumption and does so in a group who have received comparatively little attention with respect to these harms, namely midlife women. Conceptually speaking, the project is highly innovative. It tackles an issue that has troubled public health research in general for three decades – which is how to generate solutions for unhealthy behaviours that recognise the structural and systemic drivers of those behaviours, that are not reduced to programs for individual behaviour change and that collaborate with, or empower, those whose unhealthy behaviours are the focus of attention. The approach proposed, namely an application of Friere's pedagogy of the oppressed and the development and centring of critical consciousness, is highly innovative in public health research. The focus on understanding the social conditions under which unhealthy behaviour occurs is an important step, and one that standard public health methods have not easily achieved. The project quality is strengthened by combining an anti oppressive approach with social practice theory, which provides a practical framework for better understanding the paradoxes of alcohol consumption and the conditions that increase consumption, and for considering how, where and what might change. The research design has been cleverly crafted to achieve the intended project aims by bringing together a range of methodologies, notably netnography, photo elicitation, and deliberative methods alongside interviews.
However, there is tensions in the proposal between its anti-oppressive and critical / 'attuned to power' stance, and its own knowledge practices. The knowledge – of what the outcome 'should' be, of what invisible hands to look for – is held by the research team, who will author outputs, while the 'women's thought collective' (WTC) is not accorded 'advisory' status. Thus the proposal veers into sounding like the old wine of governmentality (in which 'they', the misbehaving (with consumer reference group), will optimised into willing improvement of their own behavioural governance) in new bottles of Frierian

concepts, despite the hope of achieving other-than-individual-behaviour-change actions around alcohol. A co-research design with remuneration for conference attendance and coauthoring would be a next step.

Benefit

The project is intended to produce direct benefits in the form of lower alcohol consumption in the target populations. The focus is on lower breast cancer risk, though passing mention is made of additional social benefits. The project is highly conceptually innovative and there can be no doubt that it will make a really substantial intellectual contribution to health promotion and public health research, and the concepts and approaches are likely to be widely taken up. Grappling with how to achieve action on structural and systemic drivers and how to generate real improvements in health behaviours that are also attuned to social justice goals is one of the premier challenges in public health research, and this project offers theoretical, methodological and practical tools for achieving these objectives. Even in the context of my remarks above concerning knowledge practices, participants are likely to find the process beneficial at multiple levels, and there is some opportunity to contribute to relevant policy.

Feasibility

Feasibility of this project is reasonable. The methodology has been very carefully considered and designed to answer the research questions. This project has been designed around four work packages that enable early phases of the project to inform later ones, and that distribute lead responsibility for project components across the Investigator team. The two proposed PhD students will focus on discreet project areas and will contribute substantially to the project outcomes. The budget is appropriate with personnel costing and roles in line with project requirements, noting though the gap between the remuneration of junior academic staff + teaching relief etc, and remuneration for the WTC and for women in poverty. This project proposes to do a lot in a relatively compressed space of time. The balance of budget and research focus is on the investigation of social worlds and consciousness raising work packages, and, while the design of change-steps cannot (by definition) be in any way predetermined, it is also likely to be significantly resource limited in time and financing, and may not enable more than very modest and very bounded ‘interventions’ to be trialled with fairly rudimentary evaluation.

B View

Rejoinder Status Submitted to ARC

Rejoinder Text

INVESTIGATOR
All Assessors agree wholeheartedly that the research team is “outstanding” (A) and “excellent” (B), with “exemplary” (C) track records. The capacity of the team to deliver the proposed outcomes of the study is “not in question” (B).

Assessor A comments that “the time commitment from each CI is quite small”. Time commitments noted in the application do not include the time allocation required to supervise the 2 Torrens University funded PhD students, which will increase CI Ward’s time by 0.2FTE (primary supervisor for both PhD students), from 0.25FTE to 0.45FTE and the other CIs by a further 0.1FTE each (who will co-supervise at least 1 of the PhD students); a total of 1.55FTE across the CIs which is a substantial FTE commitment.

PROJECT QUALITY AND INNOVATION
All Assessors make overwhelming positive comments, highlighting the “highly innovative” (A & C), “artfully designed” (B) and “cleverly crafted” (C) research design.

Assessor A suggests the project would benefit from a fulltime research fellow and project manager. The project includes a named postdoctoral fellow (1.0FTE for 3 years) with strong project management skills (p.86 of the budget). It also includes 2 research assistants (0.4FTE each for 2 years) to assist with data collection/analysis for 2 case-study groups at La Trobe and Adelaide Universities. Assessor A also requested explanation of project governance. Under Project Governance (p.3 of the proposal), the membership, roles and meeting schedule of the Project Management Group and Stakeholder Advisory Group are explained.

[illegible] 3 years) with strong project management skills (p.86 of the budget). It also includes 2 research assistants (0.4FTE each for 2 years) to assist with data collection/analysis for 2 case-study groups at La Trobe and Adelaide Universities. Assessor A also requested explanation of project governance. Under Project Governance (p.3 of the proposal), the membership, roles and meeting schedule of the Project Management Group and Stakeholder Advisory Group are explained.

Assessor B notes that the section describing the effectiveness of the interventions is “brief” and Assessor C that the interventions will be “trialled with fairly rudimentary evaluation”. In contrast, our approach to evaluating the interventions is also seen by Assessor C as “highly innovative”, “cleverly crafted” and one that “standard public health methods have not easily achieved”. Assessor B comments on the absence of power calculations to substantiate sample size in each intervention. With respect, the stated purpose of Work Package 3 is to assess fidelity, acceptability and effectiveness per women’s lived experiences, not measure efficacy (making sample size calculations unnecessary).

Assessor C comments that the interventions may be “modest” and “bounded” and acknowledge that these “cannot (by definition) be in any way predetermined”. Assessor A notes the interventions will be co-developed and thus “highly relevant and specific to the women.” Congruent with Participatory Action Research, a methodology which Assessor A notes we are “well versed”, we cannot specify prior to data collection what the interventions will entail; Assessor A adds the “approach is excellent.”

Assessor C comments on a perceived tension between the project’s “anti-oppressive and critical/‘attuned to power’ stance, and its own knowledge practices” and notes “the 'women's thought collective' (WTC) is not accorded 'advisory' status”. Women in the WTC are included as co-researchers and will engage in iterative and multiple levels of co-design (see p.78) throughout the project. Assessor C counters their own comment by stating “Even in the context of my remarks above concerning knowledge practices, participants are likely to find the process beneficial at multiple levels”. In addition, Assessor A highlights our co-design approach “offers a way to create interventions that, while reflective of the wider structural social worlds in which people live, respond to the needs of these women as articulated by themselves”.

BENEFIT
All Assessors only make extremely positive comments on Benefits.

All Assessors comment on the “very clearly demonstrated scholarly and practical benefits” (A), with Assessor B being “very convinced of the benefits of the study” and Assessor C having “no doubt that it [this project] will make a really substantial intellectual contribution”.

FEASIBILITY
All Assessors regard our project as highly feasible.

Assessor C questions the level of remuneration for members of the WTC. The budget includes ‘sitting fees’ per State Government guidelines (also acceptable to ethics committees per our previous studies) for the 20 WTC members across 3 years.

Although Assessor B notes this is an “ambitious project with many moving parts”, the project is “very feasible and good value for money” (A) due to: the experience and capacity of the research team (All Assessors), the 2 Torrens’ funded PhDs (“adds significantly to the ability of the CIs to deliver” (B)), that the “methodology has been very carefully considered and designed to answer the research questions” (C), the established track record of CIs & PIs working and publishing together, including our previous ARC DP (All Assessors), and that “each CI has good practice and policy experience, which positions them well to access recruits for the planned interviews and use the evidence generated to create impact” (A).
